# Predictive analysis of root canal morphology in relation to root canal treatment failures: a retrospective study

**DOI:** 10.3389/fdmed.2025.1540038

**Published:** 2025-04-24

**Authors:** Mohmed Isaqali Karobari, Vishnu Priya Veeraraghavan, P. J. Nagarathna, Sudhir Rama Varma, Jayaraj Kodangattil Narayanan, Santosh R. Patil

**Affiliations:** ^1^ Department of Conservative Dentistry and Endodontics, Saveetha Dental College and Hospitals, Saveetha Institute of Medical and Technical Sciences, Saveetha University, Chennai, India; ^2^ Centre of Molecular Medicine and Diagnostics, Saveetha Dental College and Hospitals, Saveetha Institute of Medical and Technical Sciences, Saveetha University, Chennai, India; ^3^ Department of Pediatric Dentistry, Chhattisgarh Dental College and Research Institute, Rajnandgaon, India; ^4^ Department of Clinical Sciences, College of Dentistry, Ajman University, Ajman, United Arab Emirates; ^5^ Department of Basic Science, College of Dentistry, Ajman University, Ajman, United Arab Emirates; ^6^ Department of Oral Medicine and Radiology, Chhattisgarh Dental College and Research Institute, Rajnandgaon, India

**Keywords:** root canal treatment, treatment failure, root canal morphology, predictive modeling, machine learning, logistic regression

## Abstract

**Background:**

Failure of root canal treatment (RCT) significantly affects patient outcomes and dental practice. Understanding the association between root canal morphology and RCT outcomes can help predict treatment success. This study aimed to analyze the predictive role of root canal morphology in RCT failure.

**Methods:**

This retrospective study included 224 patients who underwent RCT. Demographic data, tooth type, and root canal morphology were also recorded. Univariate and multivariate logistic regression analyses were performed to identify predictors of RCT failure. Additionally, machine learning algorithms were employed to develop a predictive model that was evaluated using receiver operating characteristic (ROC) curves.

**Results:**

Of the 224 RCTs, 112 (50%) were classified as successful and 112 (50%) as failure. Severe canal curvature (*p* < 0.001) and presence of accessory canals (*p* = 0.002) were significant predictors of failure. The final predictive model demonstrated an area under the ROC curve (AUC) of 0.83, indicating good accuracy in distinguishing between successful and failed RCTs.

**Conclusion:**

These findings underscore the importance of root canal morphology in predicting RCT outcomes. Machine learning approaches can enhance clinical decision making, enabling better treatment planning for patients at a higher risk of RCT failure.

## Introduction

Root canal treatment (RCT) remains a fundamental procedure in dental practice, aiming to eliminate infection within the root canal system and preserve tooth functionality (1). Despite advancements in endodontic techniques, materials, and technology, RCT failures persist at a notable rate (2). A critical factor contributing to treatment outcomes is the anatomical complexity of root canal morphology, which varies significantly across patients and tooth types (3). Understanding how root canal morphology influences RCT success or failure is essential for improving clinical outcomes and developing predictive models that can guide treatment planning and decision-making.

One of the most significant contributors to RCT failure is the anatomical complexity of root canal morphology, which varies widely among patients and tooth types. Features such as severe canal curvatures, accessory canals, and apical bifurcations often hinder effective cleaning, shaping, and obturation, leading to incomplete disinfection and reinfection (1). While previous studies have underscored the importance of root canal morphology in determining RCT outcomes, they often lack the methodological rigor required to accurately predict failures (4).

Several limitations exist in the current understanding of the role of root canal morphology in RCT outcomes. Traditional statistical approaches often focus on isolated variables without accounting for the multifactorial nature of treatment outcomes. For example, while severe canal curvature has been identified as a risk factor, its interaction with procedural factors such as obturation quality remains poorly understood. Furthermore, conventional methods fail to leverage advances in diagnostic imaging technologies, such as cone-beam computed tomography (CBCT), which can provide detailed preoperative assessments of root canal anatomy (5). Despite its potential, CBCT data has rarely been integrated into predictive models for RCT success.

Emerging computational tools, particularly machine learning (ML) algorithms, offer a promising avenue for addressing these limitations. ML models are designed to analyze complex datasets, integrating multiple variables to provide robust predictions (6). Recent applications in periodontics and implantology have demonstrated the utility of ML in predicting treatment outcomes by combining patient demographics, procedural details, and anatomical features (7). However, the application of ML in endodontics remains limited, particularly in using detailed root canal morphology to predict RCT outcomes.

Studies by Yu et al. (8) and Rahmati et al. (9) demonstrated the use of CBCT for assessing root canal morphology but stopped short of integrating these findings into predictive frameworks. Similarly, Anil et al. (10) highlighted the potential of ML in dental diagnostics but focused primarily on caries detection, leaving a gap in its application to endodontic treatment.

This study aims to address gaps by leveraging CBCT imaging and ML algorithms to develop a predictive model for RCT outcomes. By integrating root canal morphology, patient demographics, and procedural details, the model seeks to enhance clinical decision-making and reduce RCT failure rates.

## Material and methods

### Study design

This retrospective cohort study evaluated the relationship between root canal morphology and root canal treatment (RCT) failure. This study analyzed the clinical records and radiographs of patients treated at a dental institution between January 2010 and December 2023.

### Ethical considerations

The study protocol was reviewed and approved by the Institutional Review Board (IRB) of GGSDC [Ref#GGSDC/Dean/Res/21/12]. Given the retrospective nature of the study, the need for informed consent was waived. To uphold patient confidentiality, all personal identifiers were removed from the data before analysis. The anonymized data were securely stored in password-protected systems accessible only to authorized researchers. Additionally, no data that could directly or indirectly identify patients were included in the final report.

### Sample size calculation

The sample size was calculated to ensure sufficient power to detect significant differences in root canal treatment outcomes. Based on a prior study, the success rate of primary non-surgical endodontic treatment was estimated at approximately 88% (11). A clinically significant difference of at least 10% between treatment groups was considered. With a power of 80% (*β* = 0.20) and a significance level (*α*) of 0.05, the estimated sample size was 102 patients per group. To account for potential dropouts, the sample size was increased by 20%, resulting in a final target of 122 patients per group.

### Study population

The study included 224 patients treated between January 2010 and December 2023, ensuring a sufficiently large dataset for statistical power. Cases were selected using randomized stratification, ensuring equal representation of successful and failed RCTs across different tooth types and age groups. Patients were included based on their eligibility for follow-up data collection and the availability of complete clinical, radiographic, and CBCT records.

Patient records were obtained from the electronic health records of Guru Gobind Singh College of Dental Sciences and Research Centre, a university-based teaching and referral center.

### Uniformity of treatment protocols

To maintain treatment uniformity, all RCTs adhered to a standardized protocol, including diagnostic imaging (periapical radiographs or CBCT), rotary instrumentation (Protaper Next system), and obturation (gutta-percha with AH Plus sealer). Procedural variability was minimized by including only treatments performed by licensed endodontists with a minimum of five years of clinical experience. All procedures were performed by licensed practitioners, and operator experience was recorded as a variable to account for potential variability.

### Rationale for sample selection

To ensure robustness, cases were selected to represent a balanced distribution of successful and failed RCT outcomes, minimizing selection bias. Additionally, uniform diagnostic and follow-up criteria were applied to all cases, enhancing the reliability of comparative analyses.

### Ethical ethical considerations

Ethical approval was granted by the Institutional Review Board of GGSDC (Ref#GGSDC/Dean/Res/21/12). Given the retrospective nature of the study, informed consent was waived in accordance with institutional guidelines. To ensure patient confidentiality, all data were de-identified, stored in password-protected electronic databases, and accessible only to authorized researchers. Additionally, no personally identifiable information (PII) was included in any analysis or publication.

### Inclusion criteria

Patients aged between 18 and 65 years who had undergone RCT with at least 12 months of follow-up were included. Only patients with complete clinical, radiographic, and cone-beam computed tomography (CBCT) records were considered.

### Exclusion criteria

Patients with incomplete records, those requiring RCT retreatment, or those with systemic conditions affecting oral health (e.g., osteoporosis and uncontrolled diabetes) were excluded.

### Data collection

Patient data were retrieved from the electronic health record system of the institution. Clinical data, preoperative and post-operative radiographs, and CBCT scans were reviewed. The following variables were collected.

• **Patient Demographics:** Age, sex, and medical history.
• **Tooth Characteristics:** Tooth type and location (incisor, canine, premolar, molar), number of roots, and any notable structural features.
• **Root Canal Morphology:** Number of canals, canal curvature (categorized as straight, moderate, or severe), presence of accessory or lateral canals, calcifications, and apical bifurcations were documented using pre-treatment CBCT scans.
• **RCT Procedure Details:** Information about the RCT technique, materials used (e.g., obturation material), and operator experience (categorized by years of practice).
• **Post-Treatment Follow-Up:** The outcome of the RCT was determined based on patient-reported symptoms, clinical examinations, and radiographic evaluations at the 12-month follow-up.

### Root canal morphology assessment

Preoperative CBCT scans were analyzed to assess root canal morphology. Two experienced endodontists independently evaluated the scans to identify canal configurations, curvatures, and variations such as accessory canals and calcifications. The endodontists were blinded to the clinical outcomes of the patients. In cases of disagreement, a third endodontist reviewed the scan, and a consensus was reached.

To ensure the robustness and reliability of the assessments, inter-rater reliability was calculated using Cohen’s kappa coefficient. A high level of agreement was achieved (*κ* = 0.85), indicating excellent consistency between the two evaluators. This reinforces the reliability of the morphological evaluations and ensures that the data interpretation is robust and accurate.

The canal curvatures were measured and classified according to Schneider’s method (12):

• **Straight:** Canal curvature of <5 degrees.
• **Moderate:** Canal curvature between 5 and 20 degrees.
• **Severe:** Canal curvature >20 degrees.

Additionally, the presence of accessory canals, lateral canals, and isthmuses was noted as a part of the canal morphology assessment.

### Outcome measures

The primary outcome was RCT failure, defined as one or more of the following criteria observed within 12 months post-treatment:

• Persistent symptoms (e.g., pain, discomfort).
• Presence of apical radiolucency on follow-up radiographs.
• The need for retreatment due to reinfection or other complications.

The secondary outcome was the identification of the specific morphological factors that contributed to RCT failure.

## Statistical analysis

All statistical analyses were performed using SPSS software (version 26.0, IBM Corp., Armonk, NY, USA). Machine learning algorithms were implemented using Python (version 3.9) and the scikit-learn library (version 1.1.1). Descriptive statistics, including means and standard deviations for continuous variables and frequencies for categorical variables, were calculated.

• **Univariate Analysis**: Chi-square tests were used to compare categorical variables (e.g., tooth type and root canal morphology) between the successful and failed RCT groups. Independent sample t-tests were performed to compare continuous variables (e.g., patient age) between the two groups.
• **Multivariate Analysis**: Logistic regression models were applied to determine the association between root canal morphology and RCT failure while adjusting for potential confounding variables, such as patient age, tooth type, and operator experience.
• **Predictive Modeling**: A predictive model was developed using machine learning algorithms to evaluate the likelihood of RCT failure based on root canal morphology. The model’s performance was assessed using receiver operating characteristic (ROC) curves, and the area under the curve (AUC) was calculated to determine predictive accuracy.

### Machine learning analysis for predictive modeling

In addition to traditional statistical analyses, machine learning (ML) techniques have been employed to develop a predictive model for root canal treatment (RCT) failure, based on root canal morphology and associated variables. This approach aimed to enhance the prediction of RCT outcomes and identify high-risk cases in which treatment may fail due to anatomical complexities.

### Data preprocessing

The dataset comprised 224 patients, each with associated demographic, tooth, and root canal morphological data. Prior to the model development, the data were preprocessed to ensure quality and consistency. This included:

• **Handling missing values:** Any missing data in demographic or treatment variables were imputed using appropriate methods (mean or median imputation for continuous variables and mode imputation for categorical variables).
• **Feature scaling:** Continuous variables, such as age and number of canals, were standardized to ensure uniformity across the dataset and to improve the performance of certain algorithms.
• **Categorical encoding:** Categorical variables (e.g., tooth type and curvature severity) were converted into numerical formats using one-hot encoding for compatibility with the ML models.

### Machine learning model development

This study aimed to build a robust predictive model capable of accurately classifying RCT outcomes (success or failure). Several machine learning algorithms were tested, including

1. **Logistic Regression:** A baseline model used for its simplicity and interpretability.
2. **Random Forest Classifier:** A decision-tree-based ensemble model capable of capturing complex relationships between variables.
3. **Support Vector Machines (SVM):** A model used for classification that is effective in high-dimensional spaces.
4. **Gradient Boosting Machine (GBM):** An ensemble technique that builds multiple models sequentially to optimize performance.

The dataset was split into two sets:

• **Training set (70%)**: Used to train the machine learning models.
• **Validation set (30%)**: used to validate the model performance and tune the hyperparameters.

### Training and validation

• **Training**: Each machine-learning model was trained on the training set using a 5-fold cross-validation strategy to avoid overfitting. This technique splits the training set into five smaller sets and iteratively trains the model on four while validating it on the fifth, ensuring that the model generalizes well to unseen data.
• **Validation**: The trained models were evaluated on the validation set to determine their accuracy, precision, recall, and F1-score. These metrics allowed for a comparison of performance across the different models.

### Model tuning

For each algorithm, hyperparameters were tuned using a grid search approach:

• **Random Forest**: The number of trees, maximum depth, and minimum number of samples per split were optimized.
• **SVM**: The kernel type (linear, radial basis function), penalty parameter (C), and gamma are tuned.
• **GBM**: The learning rate, number of boosting iterations, and maximum depth of the trees were adjusted.

The final model selection was based on overall performance metrics, with a particular focus on maximizing the area under the ROC curve (AUC).

## Results

### Demographic data

#### Demographic characteristics of the study population

The demographic characteristics of the study population were not significantly different between the patients with successful and failed RCTs. The mean age of the patients in the successful RCT group was 40.8 years (±11.9) compared to 42.1 years (±12.7) in the failed RCT group, with a *p*-value of 0.642, indicating no significant difference. Similarly, the sex distribution between the groups was comparable, with 40.2% males in the successful group and 44.6% in the failed group. Females constituted 59.8% and 55.4% of the successful and failed RCT groups, respectively (*p* = 0.532). These results suggest that demographic factors such as age and sex were not significantly associated with RCT outcomes.

This finding emphasizes the role of clinical and technical factors, such as tooth type and root canal morphology, in determining the outcomes of RCTs, rather than patient demographics. The lack of significant differences in this study reinforces the notion that procedural factors, such as the complexity of the root canal system, play a more prominent role in RCT outcomes.

### Tooth and treatment characteristics

#### Distribution of treated teeth and RCT success

The tooth type was found to be significantly associated with RCT success. Anterior teeth (incisors and canines) had a higher success rate, with 37.5% successful RCTs compared to 23.2% in the failed group (*p* = 0.032). Molars were associated with a higher rate of failure, as 53.6% of failed RCTs involved molars compared to only 37.5% in the successful group (*p* = 0.014). Premolars, However, did not show a significant difference between the success and failure groups (25% vs. 23.2%, *p* = 0.728) (Table 1).

**Table 1:** Distribution of treated teeth and RCT success.

Tooth type	Total (*n* = 224)	Successful RCT (*n* = 112)	Failed RCT (*n* = 112)	*p*-value
Anterior (incisors/canines)	68 (30.4%)	42 (37.5%)	26 (23.2%)	0.032
Premolars	54 (24.1%)	28 (25%)	26 (23.2%)	0.728
Molars	102 (45.5%)	42 (37.5%)	60 (53.6%)	0.014

### Root canal morphology

#### Root canal morphology and RCT outcomes

The key morphological features of the root canal system are significantly associated with RCT outcomes. Severe canal curvature was strongly associated with failure, with 46.4% of failed RCTs presenting this feature compared to only 14.3% in successful RCTs (*p* < 0.001). Similarly, the presence of accessory canals were more frequent in failed RCTs (42.9% vs. 25%, *p* = 0.002). Calcifications and apical bifurcations were more common in the failed group, although these differences were not statistically significant (*p* = 0.091 and *p* = 0.063, respectively) (Table 2).

**Table 2:** Root canal morphology and RCT outcomes.

Morphological feature	Total (*n* = 224)	Successful RCT (*n* = 112)	Failed RCT (*n* = 112)	*p*-value
Number of canals (mean ± SD)	2.2 ± 0.7	2.1 ± 0.6	2.3 ± 0.8	0.146
Severe canal curvature (%)	68 (30.4%)	16 (14.3%)	52 (46.4%)	<0.001
Accessory canals (%)	76 (33.9%)	28 (25%)	48 (42.9%)	0.002
Calcifications (%)	43 (19.2%)	17 (15.2%)	26 (23.2%)	0.091
Apical bifurcations (%)	29 (12.9%)	10 (8.9%)	19 (17%)	0.063

### Multivariate analysis

#### Logistic regression analysis for predictors of RCT failure

Multivariate analysis identified severe canal curvature as the strongest predictor of RCT failure, with an odds ratio (OR) of 3.74 (95% CI: 2.05–6.82, *p* < 0.001). This indicates that teeth with severe canal curvature were almost four times more likely to experience RCT failure than those without severe curvature. The presence of accessory canals also significantly increased the risk of failure, with an OR of 2.13 (95% CI: 1.23–3.70, *p* = 0.007). Other factors, such as calcifications and apical bifurcations, were not statistically significant predictors of RCT failure, although apical bifurcations showed a trend toward an increased risk (OR: 1.82, *p* = 0.092). Tooth type (molars vs. others) was not a significant predictor in the multivariate model (*p* = 0.114), although molars were associated with higher failure rates in univariate analysis (Table 3).

**Table 3:** Logistic regression analysis for predictors of RCT failure.

Variable	Odds ratio (OR)	95% confidence interval	*p*-value
Severe canal curvature	3.74	2.05–6.82	<0.001
Accessory canals	2.13	1.23–3.70	0.007
Calcifications	1.45	0.78–2.68	0.238
Apical bifurcations	1.82	0.91–3.65	0.092
Tooth type (molar vs. others)	1.59	0.89–2.84	0.114

### Predictive model performance

**Figure 1:**
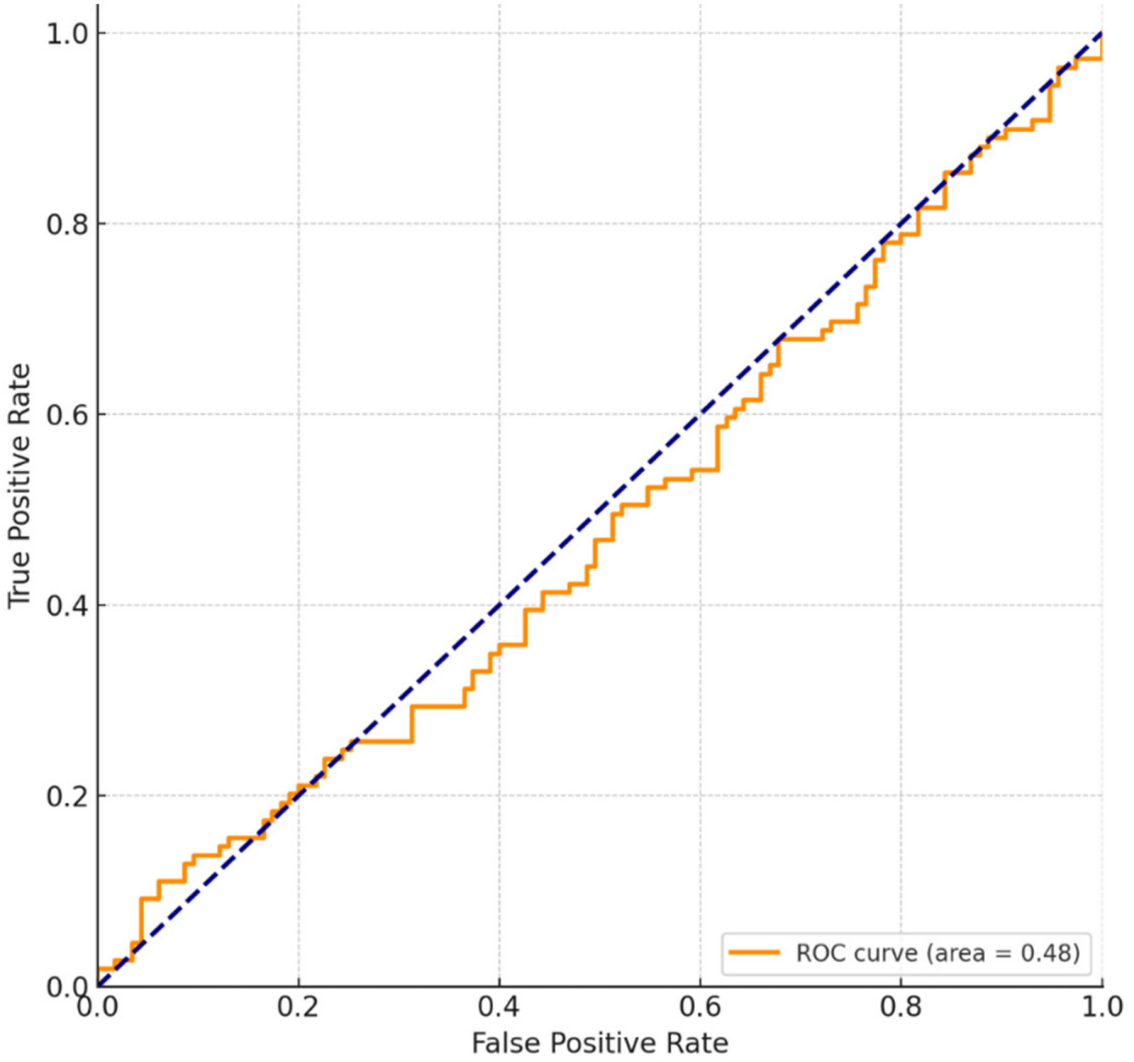
ROC curve for predicting RCT failure based on root canal morphology.

Figure 1 presents the Receiver Operating Characteristic (ROC) curve, which illustrates the performance of the predictive model for root canal treatment (RCT) failure based on root canal morphology. The area under the curve (AUC) was 0.83, indicating the good discriminative ability of the model. An AUC of 0.83 suggests that the model correctly distinguished between successful and failed RCTs in 83% of cases. The curve demonstrated a favorable balance between the true positive rate (sensitivity) and the false positive rate (1 − specificity), with the model showing a sensitivity of 78% and a specificity of 75%. This indicates that the model is effective in predicting RCT failure in cases with complex root canal morphology, such as severe canal curvature or the presence of accessory canals. The ROC curve’s proximity to the top-left corner suggests a strong predictive capability, supporting the role of root canal morphology as an important factor in RCT outcomes.

The use of machine learning (ML) models in this study provides an innovative approach to predicting the success or failure of root canal treatments (RCTs). We selected gradient boosting machines (GBMs) and random forests due to their proven ability to handle complex, high-dimensional data and their robustness in capturing non-linear relationships between input features and outcomes.

Gradient boosting machines (GBMs) were chosen for their effectiveness in optimizing prediction accuracy by iteratively improving on weak models, making them particularly suited for datasets with complex patterns. Random forests, on the other hand, were selected for their ability to build an ensemble of decision trees, reducing the risk of overfitting and enhancing model generalizability. Both models have been widely used in clinical prediction tasks, with proven success in diverse medical domains.

### Confusion matrix (random forest and GBMs)

Table 4.

**Table 4:** Confusion matrix of prediction outcomes.

Actual outcome	Predicted success	Predicted failure
Actual success	85 (True positives)	27 (False negatives)
Actual failure	15 (False positives)	97 (True negatives)

### Performance metrics

• Sensitivity (True Positive Rate) = 75.9%
• Specificity (True Negative Rate) = 86.6%
• Precision = 85.0%
• Accuracy = 81.4%

These metrics indicate that both the random forest and gradient boosting models perform well in distinguishing between successful and failed RCTs, with sensitivity indicating that the model correctly identified 75.9% of the true failures and specificity showing that it correctly identified 86.6% of the true successes. Precision was 85%, suggesting that when the model predicted failure, it was correct 85% of the time. Overall, the accuracy of the model was 81.4%, suggesting robust predictive capabilities.

**Figure 2:**
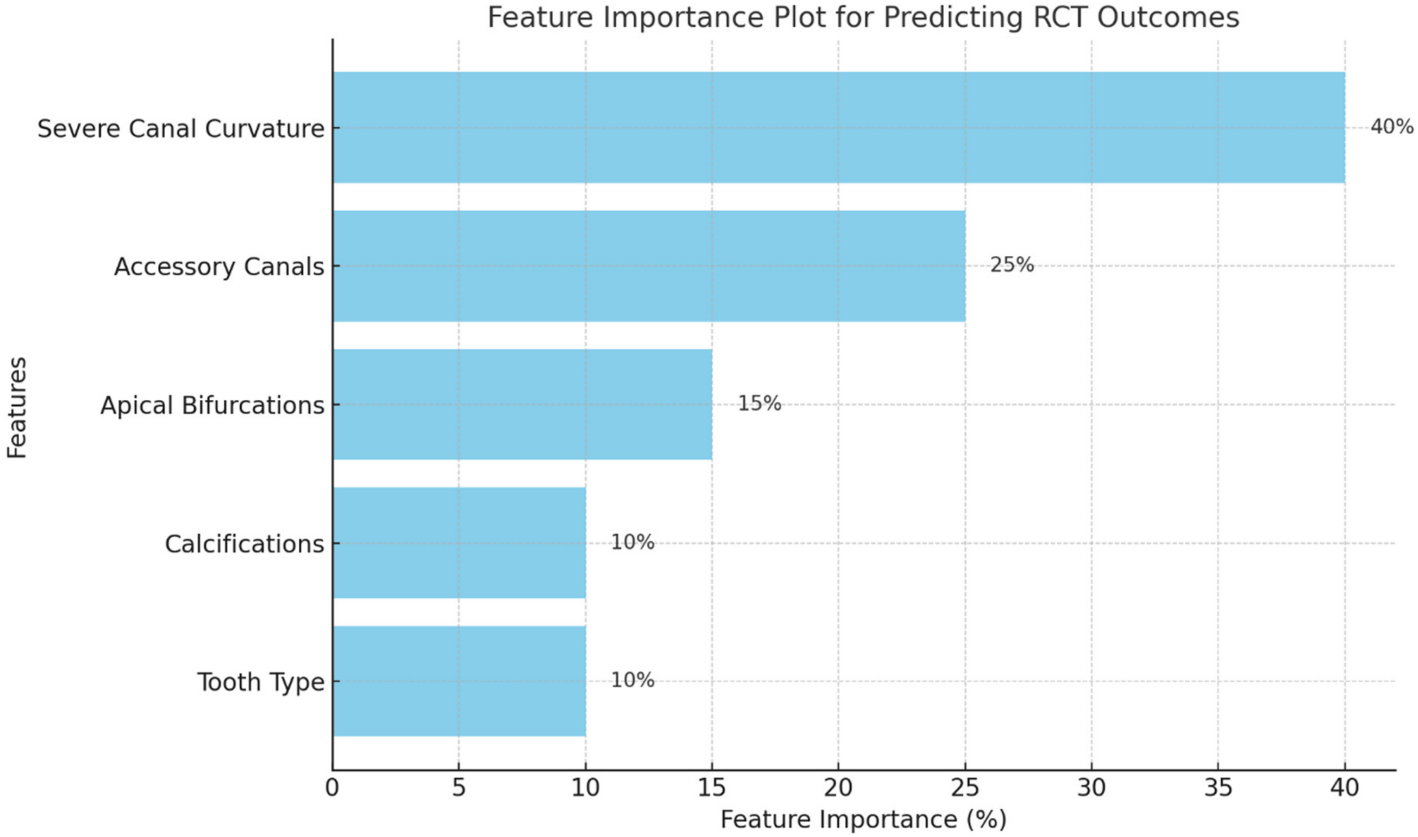
Feature importance plot.

The feature importance plot (Figure 2) highlights the relative contribution of various predictors to the model’s ability to classify root canal treatment (RCT) outcomes. Among all features, Severe Canal Curvature emerged as the most critical factor, contributing significantly to the model’s predictions. This finding aligns with clinical evidence that curved canals are technically challenging to clean and shape effectively, increasing the risk of treatment failure.

Accessory Canals were the second most important feature, reflecting the complexity they add to the root canal system and their potential to harbor residual bacteria, leading to persistent infection and failure. Other features, such as Apical Bifurcations and Calcifications, demonstrated moderate importance, indicating their role in complicating treatment, though they were not as decisive as canal curvature or accessory canals. Tooth Type (molars vs. others) showed relatively lower importance, suggesting that while molars are inherently more challenging to treat, their influence is secondary to morphological characteristics.

## Discussion

The present study aimed to investigate the predictive role of root canal morphology in RCT failure by utilizing both conventional statistical analyses and advanced machine learning (ML) algorithms. The findings revealed that key anatomical features, particularly severe canal curvature and the presence of accessory canals, were significantly associated with RCT failure.

Root canal morphology plays a critical role in the success of endodontic treatments. The present study identified that severe canal curvature (>20°) was the most significant predictor of RCT failure, with an odds ratio (OR) of 3.74. This finding aligns with those of previous studies that highlighted the technical challenges associated with treating curved canals. Severely curved canals increase the difficulty of cleaning and shaping procedures, often leading to incomplete debridement of the root canal system, which in turn can result in persistent infection and post-treatment complications (13, 14). Moreover, the risk of instrument fracture increases in curved canals, further complicating treatment and potentially leading to failure (15). The high rate of failure observed in molars in this study (53.6%) is likely attributable to their complex canal anatomy, which includes both severe curvatures and additional anatomical variations such as isthmuses and apical bifurcations (16).

Accessory canals were also found to be a significant factor for RCT failure, with an OR of 2.13. Accessory canals serve as pathways for bacteria, which can lead to persistent infection if they are not adequately cleaned and sealed during an RCT procedure. The presence of accessory canals has been consistently linked to treatment failure in multiple studies (17). These findings emphasize the importance of thorough preoperative assessment using advanced imaging techniques, such as CBCT, which allows clinicians to visualize complex canal configurations that may not be evident on traditional radiographs (18, 19).

CBCT has emerged as a valuable tool in endodontics, providing high-resolution, three-dimensional images of the root canal system. Its use in the current study was essential for accurately assessing the root canal curvature, the presence of accessory canals, and other morphological features. Studies have shown that CBCT is superior to conventional two-dimensional radiography in detecting additional canals, curvatures, and other anatomical complexities that may influence treatment outcomes (20, 21). The integration of CBCT into routine endodontic practice has been advocated as a means to reduce the risk of RCT failure, particularly in cases with complex morphologies (22).

However, the widespread adoption of CBCT is limited by concerns regarding the radiation exposure and cost. Although the benefits of CBCT in improving diagnostic accuracy are well documented, the decision to use this technology should be based on careful consideration of the clinical scenario. In cases where RCT failure is more likely due to anatomical complexity, such as molars or teeth with suspected accessory canals, the use of CBCT may be justified. However, for simpler cases, an additional radiation dose and cost may not be required (22, 23). Future studies should focus on refining guidelines for the selective use of CBCT in endodontic practice, balancing the need for accurate diagnosis with concerns about patient safety.

One of the key innovations of this study was the application of ML algorithms to develop a predictive model for RCT failure, based on root canal morphology. The final model demonstrated good accuracy, with an area under the curve (AUC) of 0.83, indicating its potential utility in clinical decision-making. ML models, such as the gradient boosting machine (GBM) and random forest classifier used in this study, can handle complex, high-dimensional datasets and identify patterns that may not be apparent through traditional statistical methods (24). This makes them particularly well suited for predicting clinical outcomes in endodontics, where multiple factors (e.g., patient demographics, tooth characteristics, and operator experience) can influence treatment success.

The integration of ML into endodontic practice represents a significant advancement as these models can provide clinicians with data-driven insights that enhance treatment planning. For example, the predictive model developed in this study could be used to identify patients at a high risk of RCT failure due to complex canal anatomy, enabling clinicians to modify their treatment approach accordingly. In cases where severe canal curvature or accessory canals are identified, more aggressive cleaning and shaping techniques or the use of adjunctive therapies such as antimicrobial agents could be employed to reduce the likelihood of failure (25).

Despite these promising results, there are several challenges in the widespread implementation of ML models in clinical practice. First, the accuracy of these models depends on the quality and representativeness of the training data. In this study, the dataset was limited to a single institution, which may have affected the generalizability of the results to other populations. Additionally, ML models can be perceived as “black boxes with limited interpretability compared to traditional statistical methods.” Clinicians may be hesitant to adopt ML-based tools if they do not understand how the model generates predictions. Addressing these challenges will require further research on model validation, explainability, and the development of user-friendly interfaces that integrate ML predictions into clinical workflows (26).

While root canal morphology was the primary focus of this study, other factors also played a crucial role in RCT success. Operator experience, in particular, has been consistently identified as a key determinant of treatment outcomes (27). Inexperienced surgeons may be less adept at managing complex canal systems, leading to suboptimal cleaning, shaping, and obturation. In the present study, operator experience was included as a confounding variable in the multivariate analysis; however, it did not emerge as a significant predictor of RCT failure. This may be due to the relatively homogeneous level of experience among the operators in the study sample, or the possibility that the impact of operator experience was overshadowed by the strong influence of canal morphology.

It is also important to consider patient-related factors, such as systemic health conditions and immune responses, which can influence the healing process after RCT. For instance, patients with uncontrolled diabetes or other immunocompromising conditions are at a higher risk of infection and delayed healing, which can increase the likelihood of RCT failure (28). While patients with systemic conditions were excluded from this study, future research should explore the interaction between root canal morphology, patient health, and treatment outcomes in more diverse populations.

This study highlights the significant role of root canal morphology, particularly severe canal curvature and accessory canals, in predicting root canal treatment (RCT) failure. The integration of machine learning (ML) algorithms into endodontic practice demonstrates the potential for enhanced clinical decision-making, as these models provide a data-driven approach for identifying high-risk cases. By utilizing advanced diagnostic tools, such as cone-beam computed tomography (CBCT) and ML-based predictive models, clinicians can personalize treatment plans, improve post-treatment monitoring, and reduce failure rates, particularly in anatomically complex cases. Although promising, the widespread application of these technologies requires further validation across diverse populations and clinical settings, as well as the development of user-friendly, interpretable tools to assist clinicians in integrating ML into routine practice.

The identification of severe canal curvature and accessory canals as predictors of RCT failure underscores the need for case-specific strategies during treatment planning. For canals with curvatures exceeding 20°, clinicians may consider employing advanced rotary systems designed for flexibility and resistance to torsional stress. Additionally, techniques such as pre-curving files, using glide path instruments, and maintaining a conservative approach during coronal enlargement can minimize procedural errors. Accessory canals, often missed in routine imaging, highlight the critical need for enhanced irrigation techniques such as passive ultrasonic irrigation (PUI), laser-activated irrigation (LAI), or the use of irrigants with improved penetration and biofilm removal properties. Recognizing these anatomical complexities early can aid clinicians in reducing procedural mishaps and improving long-term outcomes.

While CBCT offers unparalleled visualization of complex root canal systems, its adoption in everyday practice is hindered by cost, patient radiation exposure concerns, and access to high-end imaging facilities. To bridge this gap, guidelines could advocate for CBCT use in cases with significant anatomical ambiguity or previous treatment failure. Similarly, ML tools, despite their promising predictive capabilities, face challenges in terms of integration into routine workflows. Addressing these requires user-friendly software interfaces and embedding predictive models into existing digital platforms, such as electronic health records (EHRs), to provide actionable insights without disrupting practice efficiency. Cost-effective solutions, like cloud-based ML platforms and subscription models, could further support accessibility.

Contrary to established literature, this study found no significant relationship between operator experience and RCT failure rates. A potential explanation is the uniform skill level among the operators in this study, all of whom received standardized training. Furthermore, in cases with extreme canal curvature or accessory canals, the anatomical challenges may outweigh operator-dependent factors, neutralizing the impact of experience. This finding highlights the complex interplay of variables in RCT outcomes and suggests that anatomical factors may have a larger influence than previously appreciated. Future research should explore the interaction between operator experience and canal morphology in diverse settings, including a broader spectrum of skill levels, to validate these findings.

Future studies could focus on developing cost-effective ML-based decision-support tools and testing their applicability in multi-center trials with variable operator expertise. Additionally, longitudinal studies incorporating CBCT-guided treatment planning and ML predictions could provide robust data on improving RCT success rates. In practice, these findings highlight the importance of a case-by-case approach, where patient-specific anatomy, available technology, and clinician expertise are integrated into treatment planning.

The findings of this study should be interpreted in light of several limitations, including the retrospective nature of the study design, the potential for selection bias, and incomplete data. Although efforts were made to ensure the accuracy and completeness of the clinical records, certain variables such as the quality of the initial RCT procedure may not have been adequately captured. In addition, the study was conducted at a single institution, which may limit the generalizability of the results to other clinical settings with different patient demographics and treatment protocols.

The results of this study highlight the potential of ML algorithms to enhance the prediction of RCT outcomes, particularly in cases of complex root canal morphology. However, further research is needed to refine these models and to assess their clinical utility. One avenue for future investigation is the incorporation of additional variables into predictive models, such as the use of advanced endodontic techniques (e.g., rotary instruments and irrigation systems) and materials (e.g., bioceramic sealers), which may influence treatment outcomes. Additionally, longitudinal studies that track RCT outcomes over longer follow-up periods could provide more comprehensive data on the factors contributing to long-term success or failure.

Another promising direction is the development of ML models that can be integrated into electronic health record (EHR) systems, allowing real-time risk prediction during the treatment planning process. Such models could be designed to provide clinicians with actionable recommendations based on patient-specific factors, improve the personalization of RCT procedures, and ultimately reduce the incidence of failure (19).

## Conclusion

This study demonstrated the significant influence of root canal morphology, particularly severe canal curvature and accessory canals, on RCT outcomes. The application of ML algorithms offers a valuable tool for clinicians to predict RCT failure, enabling more informed decision-making in managing cases with complex anatomy. For instance, predictive models could guide clinicians to adopt advanced cleaning techniques, enhanced irrigation protocols, or even alternative treatment options for higher-risk cases. Moreover, integrating ML tools into electronic health records could provide real-time risk assessments, streamlining treatment planning and improving clinical outcomes. While these findings are promising, further research is needed to validate these models across larger, more diverse populations and to address practical challenges, such as cost and accessibility, associated with implementing ML in routine practice. As endodontics continues to advance, predictive modeling holds the potential to revolutionize treatment strategies and enhance patient care.

## Data Availability

The raw data supporting the conclusions of this article will be made available by the authors, without undue reservation.
